# Clinical decision support tools: analysis of online drug information databases

**DOI:** 10.1186/1472-6947-7-7

**Published:** 2007-03-08

**Authors:** Kevin A Clauson, Wallace A Marsh, Hyla H Polen, Matthew J Seamon, Blanca I Ortiz

**Affiliations:** 1 Nova Southeastern University, College of Pharmacy – West Palm Beach, Palm Beach Gardens, FL 33410, 3970 RCA Boulevard, Suite 7006A, USA; 2 Shenandoah University, Bernard J. Dunn School of Pharmacy, Winchester, VA, USA; 3 Health Care District of Palm Beach County, Delray Beach, FL, USA; 4 Nova Southeastern University, College of Pharmacy, Davie, FL, USA; 5 Nova Southeastern University, College of Pharmacy – Ponce, Ponce, Puerto Rico, USA

## Abstract

**Background:**

Online drug information databases are used to assist in enhancing clinical decision support. However, the choice of which online database to consult, purchase or subscribe to is likely made based on subjective elements such as history of use, familiarity, or availability during professional training. The purpose of this study was to evaluate clinical decision support tools for drug information by systematically comparing the most commonly used online drug information databases.

**Methods:**

Five commercially available and two freely available online drug information databases were evaluated according to scope (presence or absence of answer), completeness (the comprehensiveness of the answers), and ease of use. Additionally, a composite score integrating all three criteria was utilized. Fifteen weighted categories comprised of 158 questions were used to conduct the analysis. Descriptive statistics and Chi-square were used to summarize the evaluation components and make comparisons between databases. Scheffe's multiple comparison procedure was used to determine statistically different scope and completeness scores. The composite score was subjected to sensitivity analysis to investigate the effect of the choice of percentages for scope and completeness.

**Results:**

The rankings for the databases from highest to lowest, based on composite scores were Clinical Pharmacology, Micromedex, Lexi-Comp Online, Facts & Comparisons 4.0, Epocrates Online Premium, RxList.com, and Epocrates Online Free. Differences in scope produced three statistical groupings with Group 1 (best) performers being: Clinical Pharmacology, Micromedex, Facts & Comparisons 4.0, Lexi-Comp Online, Group 2: Epocrates Premium and RxList.com and Group 3: Epocrates Free (p < 0.05). Completeness scores were similarly stratified. Collapsing the databases into two groups by access (subscription or free), showed the subscription databases performed better than the free databases in the measured criteria (p < 0.001).

**Conclusion:**

Online drug information databases, which belong to clinical decision support, vary in their ability to answer questions across a range of categories.

## Background

Considering the estimate that the entire body of medical knowledge doubles every two years, it is no surprise that health information technology and computer-based decision support resources have been targeted for their potential value in enhancing safety and improving patient outcomes [1,2]. In addition to online databases that provide access to the primary literature such as Medline, commercially available databases are often used to assist with decision making. One example of resources is the online drug information databases. These drug information databases are used to assist in enhancing clinical decision support regarding a number of patient-related therapeutic choices including: determining weight-based or renally-impaired dosing regimens, monitoring for drug interactions, and identifying safety risks [3]. However, the choice of which commercial database to consult, purchase or subscribe to is likely made based on subjective elements such as history of use, familiarity, or availability during professional training. A handful of evaluations of these types of resources have been undertaken including a forward-thinking paper published in 1997 which focused on evaluating electronic databases for questions specific to decentralized pharmacists [4]. Another article looked at several drug databases from the perspective of librarians and pharmacists, but used only ten questions in their assessment and omitted some of the most commonly used databases [5]. Finally, a few recent articles have been published which examined a narrow spectrum such as electronic or online databases used for identifying prescription and over-the-counter (OTC) solid dosage forms and herb-drug interactions [6-8]. None of those articles targeted overall drug database use by healthcare practitioners nor were they comprehensive in their database selection. Thus, there is nothing in the published literature that provides a systematic and objective evaluation of the clinical decision support tool this article focused on – the online drug database. Additionally, no studies have been published comparing commercially available and freely available online drug databases.

### Objective

The objective of this study was to evaluate clinical decision support tools for drug information by systematically analyzing the most commonly used online drug information databases.

## Methods

### Database selection

There are three primary categories of online drug databases: Category 1: comprehensive; Category 2: full-text versions of an electronic book; and Category 3: freely available online databases [9]. Our primary focus was to assess the comprehensive databases that are most commonly used in a clinical decision support role by healthcare professionals. In order to determine database inclusion in the analysis, several factors were considered including: 1) usage rates in hospitals, clinics, and academic institutions, 2) previously published subjective reviews, and 3) online accessibility.

It was also determined that the online drug databases selected for inclusion should provide comprehensive information about drug therapy and contain the types of information needed across the healthcare spectrum. Additional features of the databases such as calculators, treatment algorithms, and other value-added functions would not be examined in this evaluation. Databases containing information to answer questions of particular importance to pharmacists and physicians were emphasized [10]. Five subscription databases satisfied our criteria including: Clinical Pharmacology, Epocrates Online Premium, Facts & Comparisons 4.0, Lexi-Comp Online, and Micromedex. For a secondary focus, we also selected two Category 3 (freely available) databases including Epocrates Online Free and RxList.com. Some databases that we examined have different bundles, or packages of components, available for purchase. We elected to compare the baseline bundles of all databases when applicable. The details of each database package can be found in Table 1.

### Question development

Fifteen categories of drug information questions (e.g. drug dosing, drug interactions, mechanism of action/pharmacology, side effects, and over-the-counter drug information) were identified as important to healthcare professionals based on the published literature and the Nova Southeastern University Drug Information Centers' records of queries by primary care providers. The number of questions placed in each category was weighted, with more important categories receiving more questions. For example, a category directly tied to patient safety such as drug interactions contained 17 questions, whereas the foreign drug identification category included only 7 questions. Questions were written by the authors and then reviewed by pharmacists representing different disciplines. Following feedback from the reviewers, a grand total of 158 representative questions populated the categories. Answers for the selected questions were verified against a minimum of one 'gold standard' resource such as the package insert or Physician's Desk Reference (PDR) and information located in the primary literature. Answers for questions that were not typically covered by the PDR such as off-label indications were generated from sources such as the United States Pharmacopeia and the primary literature.

The questions were used to evaluate the functionality of the databases, specifically by scope, completeness, and ease of use (EOU). Scope was assessed by the presence or absence of an answer for each question and assigned a value of one or zero accordingly. A three-point scale was used to evaluate completeness with one being least complete and three being most complete. Ease of use was measured by the number of clicks or steps necessary to reach the answer. The most direct method or shortest route to each answer was reported for ease of use. A composite score of the three facets was also generated by weighting the scope 70% and completeness 30%. Then, the EOU score was subtracted from the weighted value to determine the final score.

### Assessment techniques

All databases were evaluated by two authors in November 2005. When a discrepancy occurred in scoring, usually regarding completeness, the issue was discussed until a consensus was reached. The percentages for each evaluative component, along with mean scores and tabulated raw scores were compiled. Scope, completeness, ease of use and composite scores were all compared between databases using Scheffe's post-hoc multiple comparison test and the Chi-square test. The composite score was subjected to sensitivity analysis to investigate the effect of the choice of percentages for scope and completeness. Comparisons between subscription and free databases were also made.

## Results

### Scope

The scope evaluation component was designed to determine if a correct answer was present in the database for each corresponding question. The scores for scope are presented as a mean and a percentage for each of the fifteen question categories (Table 2). The databases able to answer the largest percentage of all 158 questions were: Clinical Pharmacology (86.7%), Micromedex (83.5%), Lexi-Comp Online (82.9%) and Facts & Comparisons 4.0 (81.0%). Pair-wise comparisons revealed three discrete tiers of database performance including: Tier 1 (Scope 128–137), Tier 2 (Scope 103 and 103) and Tier 3 (Scope 84) which were all significantly different from each other (p < .05). Databases did not get credit for providing an incorrect or misleading answer; however, they also did not suffer a penalty or negative score (such as subtracting one point from scope). There were very few cases of erroneous information in the databases.

### Completeness

Completeness was used to assess how comprehensive the database was in terms of its ability to answer each question. A three-point scale was used with a score of one indicating a cursory answer and three indicating a complete, correct answer. Many questions were structured in such a way that they would receive a completeness score of '3' if they had a scope score of '1' (e.g. What is the bioavailability of oral levofloxacin? 99%). If an answer had two components (e.g. Is lamivudine used to prevent human immunodeficiency virus (HIV) following accidental needlesticks? Yes, it is an off-label use) then completeness would be scored either a '2' or a '3'. For questions requiring three or more components to provide a complete answer, the completeness score was assigned accordingly (e.g. What is the recommended dose of lepirudin for heparin-induced thrombocytopenia? (0.4 mg/kg up to 110 kg as a bolus, then 0.15 mg/kg up to 110 kg infusion. Dose is adjusted based on an activated partial thromboplastin time (aPTT) ratio). Completeness scores were only assigned if there was a score for scope. Therefore, questions which had a score of zero for scope were not given a score of zero for completeness. The performance of the databases for completeness is reported in Table 3. Also similar to the scores for Scope, results for Completeness were stratified according to the same clustered Tier system and had the same occupants.

### Ease of use

Ease of use was designed to measure how simple, direct, and user-friendly the database would be under optimal conditions. Optimal conditions were defined as the fastest possible route from the initial database screen to the desired answer. Several other options were considered for measuring EOU based on previous evaluations such as a visual analogue scale (VAS), two comprehensive questions with Likert-scale ratings, and time (in seconds) [4,5,11]. However, in order to use a more systematic and comprehensive approach, we chose the previously employed method of the number of steps or clicks to reach the answer in order to be as objective as possible and to reduce confounders such as a 'learning curve'[12]. This direct approach may not mimic the path that inexperienced users with the databases would take, but it was deemed to be the best alternative. The mean numbers of clicks or steps were as follows: Epocrates Online Free (1.66), Epocrates Online Premium (1.72), Lexi-Comp Online (2.16), Micromedex (2.70), Facts & Comparisons 4.0 (3.02), Clinical Pharmacology (3.50), and RxList.com (3.17). Full results for ease of use are listed in Table 4. Note that the fewer the number of steps necessary, the faster and easier the information could be accessed.

### Composite Scores

In order to integrate all of the different evaluation criteria, a composite score was calculated from the scope, completeness and ease of use scores. Clinical Pharmacology earned the highest score followed by Micromedex, Lexi-Comp Online, and Facts & Comparisons 4.0. Full results are presented in Table 5. A sensitivity analysis was performed around the choice of the weighting of the scope and completeness score (70% and 30%). Varying the weighting factor to 60–40 and 50–50, did not change the ordering of the databases by composite score.

### Subscription vs. Free Online Databases

The mean scope of the subscription databases was compared to that of the free databases. The subscription databases were found to have a statistically broader scope than the free databases (p < .01).

### Errors

Of the 158 questions across the seven databases that were evaluated, there were only three cases in which the information provided by the database was different than those defined as correct by the references outlined previously for answer generation. The conflicting answers were primarily in the Dosage/Schedule section. There were two answers from RxList.com that differed with the gold standard reference. For the question: "What is the dose of potassium iodide for a 6-year-old in a radiation emergency with exposure > 5 cGy?", the database stated the dose to be 100 mg. The correct answer is 65 mg daily. The second erroneous answer provided by RxList.com fell under the OTC category, but was again a dosage related error. For the question: "What is the OTC weight-based dose of ibuprofen for a child weighing 35–47 pounds?" the database provided a chart with the answer as 100 mg every 6 to 8 hours, while the correct answer should have been 150 mg every 6 to 8 hours. The question, "What is the recommended dose of paroxetine for general anxiety disorder?" was answered by Clinical Pharmacology as "initially, 10 mg PO once daily, usually in the morning. Doses should be increased by 10 mg/day at weekly intervals if needed and tolerated. The target dose is 40 mg PO once daily and maximum dose is 60 mg/day." The answer based on the gold standard is, "the recommended starting dosage and the established effective dosage is 20 mg/day. There is not sufficient evidence to suggest a greater benefit to doses higher than 20 mg/day."

## Discussion

Healthcare professionals are constantly pressured to maintain and expand the knowledge base of their chosen specialty as well as an ever-increasing number of drug therapies – which include prescription pharmaceuticals, OTCs and dietary supplements including herbal products. Professionals must make clinical decisions and implement treatment plans integrating these therapies every day. One way to assist in that decision-making process is to employ tools such as an online drug information database. Thus, the choice of which clinical decision support tool is consulted could indirectly and directly impact patient care and outcomes. Factually correct and complete drug information that is easily accessible should be the paramount considerations in selecting an online database. Our study analyzed seven commercially available databases according to these criteria and found that the best performers included: Clinical Pharmacology, Micromedex, Lexi-Comp Online, and Facts & Comparisons 4.0. However, for the eight top categories of questions, two of the seven databases accounted for or tied all of the high scores for scope. Clinical Pharmacology scored the highest in three and tied for two high scores and Micromedex had the top score for two and tied for two. In an effort to further put the data into context, raw and composite scores were examined and tests were performed to measure for differences. While the descriptive statistics indicated a top performer and a rank order to all 8 databases, direct comparisons produced 3 groupings (tiers) of databases that were statistically different. All databases within a tier were found to be statistically similar.

However, given the significant difference in cost between databases, a finding of similarity is very significant. For many individuals and institutions, cost is part of the inherent value of these types of tools along with the anticipated number of users and format availability such as personal digital assistants (PDAs). For example, Micromedex and Clinical Pharmacology both include abridged PDA versions of their databases as part of their base institutional subscription package whereas Lexi-Comp Online does not.

In order to judge which online database would be the best match for an individual practitioner or institution, additional criteria should be considered. One way to utilize the results from this study is to look at how each database scored in specific categories and combine those results with practice-specific priorities. For example, some databases did particularly well (or poorly) with safety-focused questions, questions about dietary supplements, or questions reflective of a diverse patient population such as foreign drug identification. Over-the-counter drug considerations is another category in which there was a lot of variability, with scores ranging from three to eleven out of a possible thirteen. Despite being overlooked in medical histories, as treatment options, and as offending or causative agents in side effects and interactions, some databases still fail to include OTC information [13-15].

### Limitations

Most of the databases evaluated offer additional value-added functions and references beyond the baseline drug information that was measured in this study. Some have extensive calculator functions, diagnostic criteria and tools, patient education components, and formulary information. None of them were assessed in this study. Those additional components may have a direct impact on the decision-making process when selecting an online database. However, it was outside the intended scope to include them in this evaluation.

All of the databases that were analyzed in this study are updated and changed with varying frequencies. Thus, the information that was present at the time of the evaluation for each database may not be true now or in the future. This analysis represents a snapshot of the quality and accessibility of drug information each database provided at the time of the study.

The ease of use criteria certainly provides guidance for which databases are the most user-friendly; however, another element must be considered when examining the scores. The number of clicks or steps to find the answer may not take everything into account for the total time to retrieve an answer. Once the target section of the drug monograph had been reached, credit was given to the database. Therefore, a database that requires a fewer number of clicks but then requires considerable scrolling would score better than one that required more clicks but took you directly to the desired information. In practice, the total time devoted to each could be very similar. For example, an answer was located within Micromedex in an average of 2.70 steps. However, Micromedex provided the drug monograph in its entirety so the user must scroll through the monograph to locate the desired answer, or use the quick links to jump to a specific section within the monograph, thus adding steps to the process. Clinical Pharmacology scored an average of 3.50 steps to reach the desired answer. With this database, each click narrows the information provided and takes the user to a specific section of the monograph, rather than the monograph in its entirety. While one method of data organization and retrieval is not necessarily better than another, it should be considered when choosing a database. The amount of data the user must review before actually finding the desired information can greatly influence the speed of retrieval and overall utility of the database. Despite the limitations, the authors maintain that the ease of use criteria remains the most consistent and most easily reproducible method of the choices available.

We included a robust number of questions, especially relative to other evaluative studies with a similar structure; to further differentiate between databases would require a larger sample of questions [5,10,11]. In support of this possibility, it is notable that most of the widest margins between databases occurred when there were categories with 10 or more questions. We also acknowledge that an evaluation conducted with an entirely different set of questions could result in different findings.

## Conclusion

The online drug information databases we have evaluated and which belong to clinical decision support vary in their ability to answer questions across a range of categories. Ranked according to composite score, Clinical Pharmacology, Micromedex, Lexi-Comp Online, and Facts & Comparisons 4.0 were the top ranked online information databases. Additionally, the databases that require a subscription outperformed the free online databases.

## Competing interests

KAC received financial support for dissemination of results, including the article processing fee, from Elsevier Science/Gold Standard, Inc. which produces Clinical Pharmacology. The support was made available after the results of the study were already listed online in conjunction with a conference presentation.

## Authors' contributions

KAC conceived the study, contributed to the study design, assisted with data collection and drafted the manuscript; WM assisted with study design and performed the statistical analysis; HP assisted with the study conception, revised the draft manuscript, and assisted with data collection; MJS assisted with the study conception and data collection; BIO assisted with data collection. All authors contributed to and provided approval for the submitted manuscript.

## Pre-publication history

The pre-publication history for this paper can be accessed here:



## Figures and Tables

**Table 1:** Baseline Bundles and Components Evaluated

Subscription Databases:	
**Clinical Pharmacology**	Drug Information Database and Drug Interaction Analyzer
**Epocrates Online Premium**	Epocrates drug information database content plus dietary supplement information, pill identifier, patient information
**Facts & Comparisons 4.0**	Drug Facts & Comparisons, Drug Interaction Facts, The Review of Natural Products, Nonprescription Drug Therapy, MedFacts, Drug Interaction Facts: Herbal Supplements and Food, Drug Identification Tool, A to Z Drug Facts
**Lexi-Comp Online**	Lexi-Drugs, Pediatric Lexi-Drugs, Geriatric Lexi-Drugs, Natural Products, Pharmacogenomics, Infectious Diseases, Poisoning and Toxicology, Nuclear, Biological and Chemical Agent Exposures, Laboratory Tests
**Micromedex**	DRUGDEX (Drug Evaluations, Drug Consults), Identidex
Free Databases:	
**Epocrates Online Free**	Epocrates drug information database content
**RxList.com**	Internet site with information sourced from WebMD and FirstDataBank

**Table 2:** Scope of Databases

**Scope Score**	**n**	**CP**	**EP**	**FC**	**LC**	**MM**	**EF**	**RL**
**Dosage/Schedule**	17	**16**	13	14	13	15	12	11
**Drug Interactions**	17	14	13	14	13	**15**	12	12
**Methods of Administration**	13	12	12	12	12	**13**	10	12
**Adverse Drug Reactions**	13	**13**	9	12	12	**13**	9	10
**Over-the-Counter**	13	**11**	4	**11**	9	6	4	3
**Indications/Unlabeled Uses**	11	**11**	6	8	10	10	6	5
**Identification/Content**	10	**10**	7	8	9	8	5	8
**Pharmacokinetics**	10	8	5	**9**	8	**9**	3	6
**Compatibility/Stability**	9	8	4	6	**9**	8	1	6
**Contraindications**	9	**9**	6	8	7	**9**	5	8
**Dietary Supplements**	8	**8**	7	**8**	6	**8**	0	5
**Pharmacology/MOA**	8	**8**	7	**8**	**8**	7	7	**8**
**Pregnancy and Lactation**	8	7	6	**8**	**8**	**8**	6	**8**
**Foreign Drug Identification**	7	1	0	1	**3**	**3**	0	0
**Cost and Economics**	5	1	**4**	1	**4**	0	**4**	1
								
**Totals**	158	**137**	103	128	131	132	84	103
**(%)**		**86.7**	65.2	81.0	82.9	83.5	53.2	65.2

n = number of questions per category, CP = Clinical Pharmacology, EP = Epocrates Premium, FC = Facts & Comparisons 4.0, LC = Lexi-Comp Online, MM = Micromedex, EF = Epocrates Free, RL = RxList

**Table 3:** Completeness of Databases

**Completeness Score**	**CP**	**EP**	**FC**	**LC**	**MM**	**EF**	**RL**
**Dosage/Schedule**	2.88	2.92	2.93	2.92	3.00	2.92	3.00
**Drug Interactions**	2.93	2.69	2.79	2.77	3.00	2.67	2.42
**Methods of Administration**	2.92	2.67	2.92	3.00	2.92	2.60	2.58
**Adverse Drug Reactions**	2.69	2.89	2.92	2.92	3.00	2.89	2.40
**Over-the-Counter**	2.73	2.75	2.64	2.89	2.17	2.75	2.33
**Indications/Unlabeled Uses**	3.00	2.67	3.00	3.00	3.00	2.67	3.00
**Identification/Content**	2.90	2.86	2.88	2.89	2.88	2.80	2.88
**Pharmacokinetics**	2.88	2.60	2.89	2.63	2.67	3.00	2.50
**Compatibility/Stability**	2.88	2.75	2.83	2.89	3.00	3.00	2.67
**Contraindications**	3.00	3.00	3.00	2.86	2.89	3.00	2.75
**Dietary Supplements**	3.00	2.86	2.88	2.33	3.00	NA	2.80
**Pharmacology/MOA**	2.88	2.71	3.00	2.63	3.00	2.71	3.00
**Pregnancy and Lactation**	3.00	2.83	3.00	3.00	2.88	2.83	2.63
**Foreign Drug Identification**	3.00	NA	1.00	3.00	3.00	NA	NA
**Cost and Economics**	3.00	3.00	3.00	3.00	NA	3.00	3.00
							
**(%)**	96.4	93.2	95.8	95.2	**97.0**	93.2	89.6

CP = Clinical Pharmacology, EP = Epocrates Premium, FC = Facts & Comparisons 4.0, LC = Lexi-Comp Online, MM = Micromedex, EF = Epocrates Free, RL = RxList, NA = no score/no answer present

**Table 4:** Ease of Use of Databases

**Ease of Use Score**	**CP**	**EP**	**FC**	**LC**	**MM**	**EF**	**RL**
**Dosage/Schedule**	3.94	1.54	3.00	2.00	2.80	1.58	3.09
**Drug Interactions**	4.07	2.77	3.00	2.31	2.40	2.92	3.42
**Methods of Administration**	3.00	1.33	3.00	2.00	2.54	1.30	3.25
**Adverse Drug Reactions**	3.62	1.78	3.00	2.00	2.69	1.78	3.00
**Over-the-Counter**	4.25	1.75	3.00	2.00	3.00	1.75	4.33
**Indications/Unlabeled Uses**	3.55	1.50	3.00	2.00	2.90	1.50	3.00
**Identification/Content**	3.10	1.43	1.88	2.44	2.38	1.40	1.63
**Pharmacokinetics**	2.63	1.60	3.44	2.00	2.89	1.33	3.00
**Compatibility/Stability**	3.50	2.25	3.17	2.00	2.75	1.00	3.00
**Contraindications**	3.33	1.33	3.00	2.33	2.78	1.20	2.88
**Dietary Supplements**	3.25	2.29	3.00	2.67	2.25	NA	3.00
**Pharmacology/MOA**	2.00	1.00	3.00	2.00	3.00	1.00	3.00
**Pregnancy and Lactation**	3.00	1.17	3.00	2.00	2.75	1.33	3.00
**Foreign Drug Identification**	4.00	NA	3.00	2.00	3.33	NA	NA
**Cost and Economics**	14.00	2.0	10.00	3.75	NA	2.00	20.00
							
**Mean number of steps required**	3.50	1.72	3.02	2.16	2.70	**1.66**	3.17

CP = Clinical Pharmacology, EP = Epocrates Premium, FC = Facts & Comparisons 4.0, LC = Lexi-Comp Online, MM = Micromedex, EF = Epocrates Free, RL = RxList, NA = no score/no answer present

**Table 5:** Composite Scores of Online Drug Databases

**Composite Score**	**CP**	**EP**	**FC**	**LC**	**MM**	**EF**	**RL**
**Mean Scope**	**86.7**	65.2	81.0	82.9	83.5	53.2	65.2
**Mean Completeness**	96.4	93.2	95.8	95.2	**97.0**	93.2	89.6
**Scope (0.7) + Completeness (0.30)**	**89.6**	73.6	85.4	86.6	87.5	65.2	72.5
**Mean number of steps - 1**	2.50	0.70	2.02	1.16	1.75	**0.55**	2.17
**Scope (0.7) + Completeness (0.30) - (Ease of Use - 1) = Composite Score**	**87.1**	72.9	83.4	85.4	85.8	64.5	70.3

CP = Clinical Pharmacology, EP = Epocrates Premium, FC = Facts & Comparisons 4.0, LC = Lexi-Comp Online, MM = Micromedex, EF = Epocrates Free, RL = RxList
